# Quercetin Caused Redox Homeostasis Imbalance and Activated the Kynurenine Pathway

**DOI:** 10.3390/biology9080219

**Published:** 2020-08-10

**Authors:** Oluyomi Stephen Adeyemi, Chinemerem Ebugosi, Oghenerobor Benjamin Akpor, Helal F. Hetta, Sarah Al-Rashed, David Adeiza Otohinoyi, Damilare Rotimi, Akinyomade Owolabi, Ikponmwosa Owen Evbuomwan, Gaber El-Saber Batiha

**Affiliations:** 1 Department of Biochemistry, Medicinal Biochemistry, Nanomedicine & Toxicology Laboratory, Landmark University, Omu-Aran 251101, Nigeria; ebugosi.chinemerem@lmu.edu.ng (C.E.); rotimi.damilare@lmu.edu.ng (D.R.); 2 Department of Microbiology, Landmark University, Omu-Aran 251101, Nigeria; akpor.oghenerobor@lmu.edu.ng (O.B.A.); akinyomade.owolabi@lmu.edu.ng (A.O.); evbuomwan.ikponmwosa@lmu.edu.ng (I.O.E.); 3 Department of Medical Microbiology and Immunology, Faculty of Medicine, Assiut University, Assiut 71515, Egypt; helal.hetta@uc.edu; 4 Department of Internal Medicine, University of Cincinnati College of Medicine, Cincinnati, OH 45267-0595, USA; 5 Department of Botany and Microbiology, College of Science, King Saud University, Riyadh 11451, Saudi Arabia; salrashed@ksu.edu.sa; 6 College of Medicine, All Saints University, Belair VC0282, Saint Vincent and the Grenadines; adeizadavid@gmail.com; 7 Department of Pharmacology and Therapeutics, Faculty of Veterinary Medicine, Damanhour University, Damanhour 22511, AlBeheira, Egypt; gaberbatiha@gmail.com

**Keywords:** antimicrobials, microbial infection, medicinal biochemistry, polyphenols, natural products

## Abstract

The search for new and better antimicrobial therapy is a continuous effort. Quercetin is a polyphenol with promising antimicrobial properties. However, the understanding of its antimicrobial mechanism is limited. In this study, we investigated the biochemical mechanistic action of quercetin as an antibacterial compound. Isolates of *Bacillus subtilis, Pseudomonas aeruginosa, Escherichia coli, Klebsiella pneumonia,* and *Staphylococcus aureus* were initially exposed to quercetin for antibacterial evaluation. Subsequently, *S. aureus* (Gram-positive) and *E. coli* (Gram-negative) cells were exposed to quercetin with or without ascorbic acid, and cells were harvested for selected biochemical assays. These assays included redox homeostasis (lipid peroxidation, total thiol, total antioxidant capacity), nitric oxide, and kynurenine concentration as well as DNA fragmentation. The results revealed that quercetin caused lipid peroxidation in the bacterial isolates. Lipid peroxidation may indicate ensuing oxidative stress resulting from quercetin treatment. Furthermore, tryptophan degradation to kynurenine was activated by quercetin in *S. aureus* but not in *E. coli*, suggesting that local L-tryptophan concentration might become limiting for bacterial growth. These findings, considered together, may indicate that quercetin restricts bacterial growth by promoting oxidative cellular stress, as well as by reducing the local L-tryptophan availability by activating the kynurenine pathway, thus contributing to our understanding of the molecular mechanism of the antimicrobial action of quercetin.

## 1. Introduction

The rising antibiotic resistance, and consequently, the scarcity of new antimicrobials is a growing public health concern [1,2]. This challenge underscores the search for new classes of antimicrobials from natural products [3]. Plant-derived phytochemical compounds such as polyphenols have been attracting attention as potential antimicrobial substances, and the use of polyphenolic compounds may prove beneficial in increasing the susceptibility of resistant pathogens to existing antibiotics [4,5,6].

Polyphenols are ubiquitous secondary metabolites widely distributed in plants. Polyphenols are involved in health-promoting activities, including the prevention of certain cancers, diabetes mellitus, neurodegenerative diseases, cardiovascular disease, and osteoporosis [4]. These activities have been attributed initially to the free radical scavenging, antioxidant, and metal-chelating properties of these compounds. Polyphenolic compounds may also increase the susceptibility of resistant pathogens to existing antibiotics [5,6]. The potent antioxidant activity of polyphenols even results in the growth inhibition of pathogenic microorganisms [7]. Tea polyphenols are also known to have an excellent antimicrobial effect against organisms such as *Staphylococcus aureus, C. sakazakii, Serratia marcescens,* and *Salmonella enteritidis* [8].

Quercetin is a polyphenol or flavonoid abundantly found in many ethnic plants, especially tea and onion. Quercetin (4H-1-benzopyran-4-one, 2-(3,4-dihydroxyphenyl)-3,5,7-trihydroxy-flavon), also known as a glycoside, is found in different parts of plants including fruits, leaves, and flowers, and it is the most abundant type of dietary and bioactive flavonoid [9]. Quercetin is also present in some medicinal plants, including *Hypericum perforatum*, *Ginkgo biloba,* and *Sambucus Canadensis*, and is reported to exhibit diverse therapeutic effects. Quercetin, like other polyphenols, has many pharmacological activities. These activities include antioxidant, antimicrobial, cardiovascular, hepatoprotective, neurological, antiviral, anti-inflammatory, anti-cancer, and anti-obesity properties [8,9,10,11]. Quercetin also inhibits the growth of various bacterial isolates including *Escherichia coli* and *Staphylococcus aureus* with a minimum inhibitory concentration (MIC) range between 20–400 mg/mL [12]. Likewise, some studies reported on the antimicrobial activities of quercetin in combination with other agents resulting in remarkable synergistic interactions against many pathogens [8,13,14,15]. Furthermore, the bacteriostatic ability of quercetin makes it an attractive choice for antibacterial drug research. Although quercetin has prospects as an alternative antimicrobial, the understanding of the mechanism of the antimicrobial action remains limited.

It is thus essential to elucidate the molecular mechanisms of this therapeutic action of quercetin to better understand its health effects, which is the aim of this study.

## 2. Materials and Methods

### 2.1. Biochemical Reagents

The reagents used in this study were analytical grade reagents used as supplied except otherwise stated.

### 2.2. Microbial Culture

Stock cultures of selected Gram-positive and negative bacterial isolates were generated as reported elsewhere [16,17]. The purity of the strains was determined by streaking on nutrient agar plates that were incubated at 37 °C for 24 h. Only plates that showed pure colonies were sub-cultured and used for further studies. For the isolation of the pure clones, 0.5 mL of overnight cultures of the respective bacteria were inoculated in 100 mL of nutrient broth (HiMedia, Mumbai, India) before dispensing onto agar slants. The bacterial isolates included *Bacillus subtilis, Pseudomonas aeruginosa, Escherichia coli, Klebsiella pneumonia,* and *Staphylococcus aureus*. The agar slants were stored at 4 °C ± 2 °C for further studies.

#### Quercetin’s Antibacterial Evaluation and Determination of the Minimum Inhibitory Concentration (MIC)

The agar diffusion method, as previously described [16,17], was used. In this assay, nutrient agar was prepared into 100 mL capacity conical flask with 50 mL nutrient medium, as outlined by the manufacturer. The agar was sterilized for 15 min at 121 °C and allowed to cool to about 40 °C. After cooling at room temperature, 1 mL of a broth culture of each test organism was inoculated into separate flasks containing sterile media and mixed thoroughly. About 20 mL of the inoculated media were then dispensed into Petri dishes and allowed to solidify. Subsequently, holes were bored on the solidified agar using a sterile cork-borer, and the holes were filled with ascorbic acid, rutin or quercetin (Sigma, St. Louis, Missouri, USA) at 1000 µg/mL in dimethyl sulfoxide (DMSO) before incubating at 37 °C overnight. Amoxicillin antibiotics served as a reference drug as well as for assay validation. DMSO alone was used as the control. The zone of clearance was measured using a metric ruler as previously described [16,17].

The minimum inhibitory concentration (MIC) was determined by the broth dilution method described elsewhere [16]. Briefly, different bacterial strains initially grown on medium for 18 to 24 h at 37 °C were incubated with varying concentrations of quercetin (0–1000 µg/mL) for 24 h at 37 °C. At termination of the incubation, the tubes were checked for turbidity (optical density 600 nm) as evidence of growth. The lowest concentration of quercetin at which no bacterial growth occurred was taken as the MIC. All experiment setups were done in duplicate. The organisms tested were *Bacillus subtilis*, *Pseudomonas aeruginosa*, *Escherichia coli*, *Klebsiella pneumonia*, and *Staphylococcus aureus.*

### 2.3. Treatments of Cells for Biochemical Assays

In this study, *S. aureus* (Gram-positive) and *E. coli* (Gram-negative) were chosen for further biochemical experiments: 1 mL of overnight liquid cultures of each bacterial strain was inoculated into 100 mL of sterile media containing the respective treatments. The treatment protocols are presented below.

#### 2.3.1. Quercetin Treatment Only

The bacterial strains were treated at 1× MIC, 2× MIC, and 3× MIC. For *S. aureus*, quercetin at 700 µg/mL, 1400 µg/mL, and 2100 µg/mL was used, while the *E. coli* treatment was 300 µg/mL, 600 µg/mL, and 900 µg/mL.

#### 2.3.2. Quercetin and Ascorbic Acid Treatment

Simultaneous treatment with quercetin (as described above) and ascorbic acid (1000 µg/mL) was performed. To determine whether oxidative stress was part of the mechanistic action of quercetin, an antioxidant (ascorbic acid), was added. Initially, we established that this antioxidant compound at 1000 µg/mL showed no inhibition of bacterial growth.

### 2.4. Harvesting Cells for Biochemical Analysis

All treated cells were incubated at 37 °C for 24 h and harvested by centrifugation [16,17,18]. The pellet was rinsed three times with physiological saline, re-suspended, homogenized, and used for biochemical studies.

#### Biochemical Analyses

Where appropriate, biochemical assays were performed by a spectrophotometric method using a UV/Vis spectrophotometer (Jenway, Staffordshire, United Kingdom). The test for kynurenine followed an established protocol [19]. Methods to determine the nitric oxide level (nitrite/nitrate concentration) and the total antioxidant capacity (TAC) are described elsewhere [20]. A modification of the method described by Gornall et al. [21] was used to assess the total protein level. In the protein assay, the biuret reagent contained potassium iodide to avoid Cu^2+^ ion precipitation. The tests for total thiol and lipid peroxidation (malondialdehyde-MDA) were described previously [22]. DNA fragmentation was assessed by the diphenylamine (DPA) protocol [23].

### 2.5. Statistical Analysis

The data were analyzed by one-way analysis of variance (ANOVA) on GraphPad Prism 6 (San Diego, CA, USA), and the results are presented as the mean of independent experiments replicated twice with the corresponding SEM (standard error of the mean). Tukey’s post-hoc test was used to compare mean values among groups, and statistical significance was considered at *p* < 0.05. 

## 3. Results

### 3.1. Antibacterial Determination

Treatment with quercetin affected the growth of all five organisms as estimated by the zone of clearance when compared to the reference drug (i.e., Amoxicillin (Table 1)). The quercetin MIC was determined to be 300 and 700 µg/mL against *E. coli* and *S. aureus,* respectively.

### 3.2. Biochemical Assays

**Figure 1:**
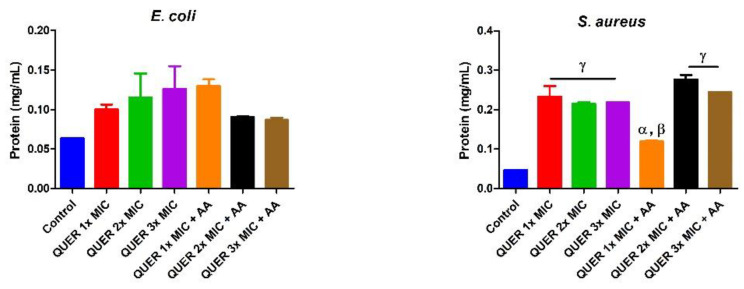
Protein concentration in bacterial isolates following treatment with quercetin (QUER) alone or combined with ascorbic acid (AA). Results are presented as an average of duplicate biological experiments (n = 2) with the corresponding standard error of the mean (SEM). Compared with the control, α at *p* < 0.05 and γ at *p* < 0.0001 are significant, while β is significant at *p* < 0.01 relative to QUER 1× MIC.

**Figure 2:**
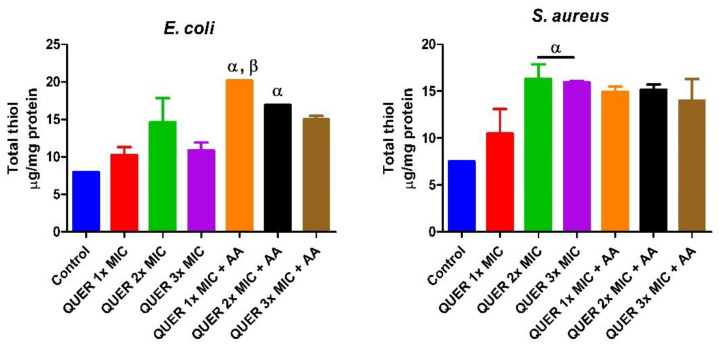
Concentration of total thiol in bacterial isolates following exposure to quercetin (QUER) alone or when combined with ascorbic acid (AA). Results are presented as an average of duplicate biological experiments (n = 2) with the corresponding standard error of the mean (SEM). Compared with the control or QUER 1× MIC, α at *p* < 0.05 is significant, and β is significant at *p* < 0.01.

**Figure 3:**
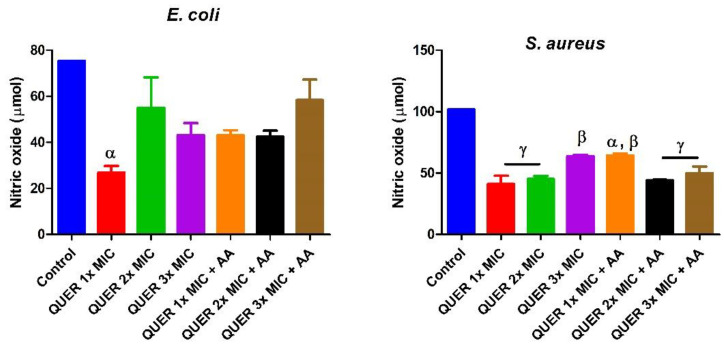
Concentration of nitric oxide in bacterial isolates following exposure to quercetin (QUER) alone or when combined with ascorbic acid (AA). Results are presented as an average of duplicate biological experiments (n = 2) with the corresponding error of the mean (SEM). Relative to the control or QUER 1× MIC, α at *p* < 0.05 is significant, β at *p* < 0.01 and γ at *p* < 0.0001.

**Figure 4:**
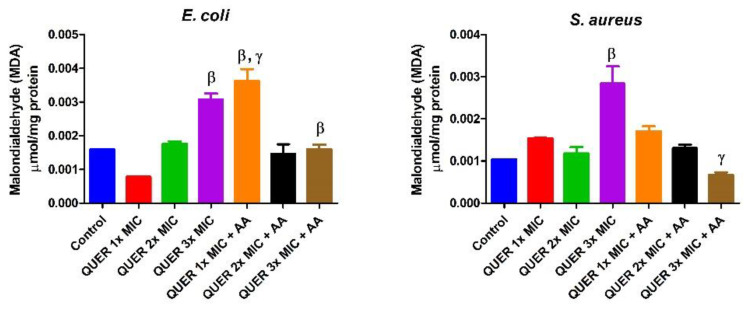
Malondialdehyde concentration in bacterial isolates following exposure to quercetin (QUER) alone or when combined with ascorbic acid (AA). Results are presented as an average of duplicate biological experiments (n = 2) with the corresponding standard error of the mean (SEM). Compared with control or QUER 3× MIC, β at *p* < 0.01 is significant and γ at *p* < 0.0001 compared with QUER 1× MIC or QUER 3× MIC.

**Figure 5:**
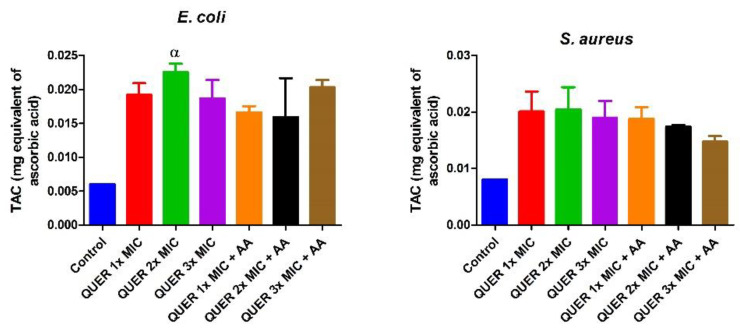
The total antioxidant capacity (TAC) of bacterial isolates following exposure to quercetin (QUER) alone or when combined with ascorbic acid (AA). Results are presented as an average of duplicate biological experiments (n = 2) with the corresponding standard error of the mean (SEM). Relative to the control, α at *p* < 0.05 is significant.

**Figure 6:**
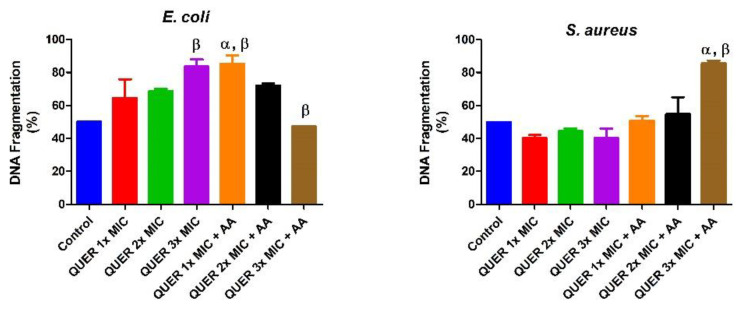
DNA fragmentation in bacterial isolates following exposure to quercetin (QUER) alone or when combined with ascorbic acid (AA). Results are presented as an average of duplicate biological experiments (n = 2) with the corresponding standard error of the mean (SEM). Relative to the control or QUER 1× MIC, α at *p* < 0.05 is significant, as is β at *p* < 0.01 when compared with the control or QUER 3× MIC.

**Figure 7:**
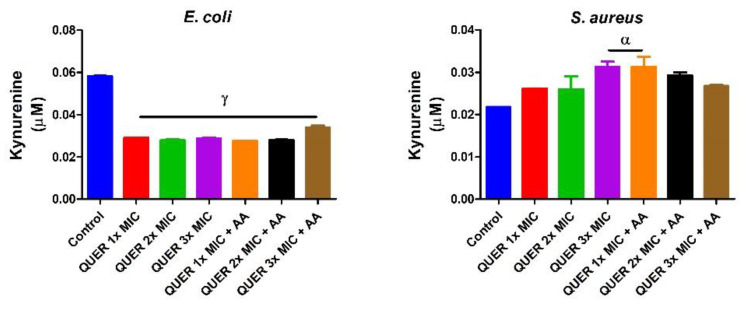
Kynurenine concentration in bacterial isolates following exposure to quercetin (QUER) alone or when combined with ascorbic acid (AA). Results are presented as an average of duplicate biological experiments (n = 2) with the corresponding standard error of the mean (SEM). Compared with the control or QUER 1× MIC, α at *p* < 0.05 is significant, as is γ at *p* < 0.0001 relative to the control.

Quercetin with or without ascorbic acid led to an increase (*p* < 0.05) in the total protein concentration of the microorganisms compared with the control. Although the protein content increased with increasing MIC concentration of quercetin only in groups against *E. coli*, the combination of quercetin and ascorbic acid treatment resulted in the highest protein level in both bacterial lysates. However, increasing the concentration of quercetin beyond the MIC in conjunction with ascorbic acid resulted in a lower protein level in *E. coli* with an opposite effect seen in *S. aureus* (Figure 1). The effect of quercetin on the total thiol levels of both bacterial strains showed an increase relative to the control. There was also noticeable potentiation between quercetin and ascorbic acid on the levels of total thiol, *p* < 0.01 in both organisms, with contrasting effects seen with the quercetin-only treatment in *E coli* (Figure 2). Quercetin and ascorbic acid also reduced the level of nitric oxide in both microbes (Figure 3). The assay for lipid peroxidation showed that the MDA level increased in both microorganisms following treatment with quercetin *p* < 0.05 (Figure 4). The TAC increased in the organisms in response to the quercetin treatment only as well as a combination with ascorbic acid *p* < 0.05 (Figure 5); this result supports the antioxidant properties of quercetin and ascorbic acid. Also, treatment with quercetin and ascorbic acid caused mild DNA damage and was more pronounced in *S. aureus* treated with a combination of quercetin and ascorbic acid (Figure 6). Furthermore, when compared with the control, kynurenine levels were increased in *S. aureus* but decreased in *E. coli* (Figure 7).

## 4. Discussion

Quercetin is reported to inhibit Gram-positive and Gram-negative bacterial proliferation by inactivating extracellular proteins [20]. However, studies on the mechanism of action of quercetin as an antimicrobial are limited. In our study, we investigated the biochemical and antimicrobial effects of quercetin on *S. aureus* (Gram positive) and *E. coli* (Gram negative). Similar to previous reports [24], quercetin had an antibacterial effect on the growth of the microorganisms, which was more pronounced in *E. coli*. This observation contrasts other studies in which *S. aureus* was more susceptive to quercetin than *E. coli* [25]. We presume that the thin peptidoglycan layer and the ability of quercetin to initiate the peroxidation of the outer lipid membrane increase the sensitivity of *E. coli* to quercetin compared with *S. aureus*. Perhaps, similar to other flavonoids, quercetin may initiate bacteriostatic properties by compromising the bacterial cell membrane integrity, thereby lysing the cell [25]. This point is supported further by the high levels of MDA noticed in this study, indicating oxidative stress when compared with the control samples. The high protein levels of treated cells compared with the control may indicate an adaptive mechanism of the bacterial cells in response to stress likely imposed by quercetin exposure. Similarly, quercetin, because of its antioxidant property, might have raised the levels of total thiol and thus increased TAC, while reducing NO levels compared to the control. Quercetin (3,5,7,3’,4’-pentahydroxyflavone) has three major structural groups that aid its antioxidant property. The functional groups including the 4-oxo group in conjugation with the 2,3-alkene, B ring *o*-dihydroxyl groups, and the 3- and 5-hydroxyl groups could donate electrons to the rings, which in turn increases the resonance forms, which is separate from the antioxidant property of the benzene structure [26]. Therefore, the increase in the total thiol concentration and TAC could be due to the antioxidant nature of not only quercetin but also ascorbic acid.

Furthermore, we observed that the action of quercetin might not preclude the modulation of tryptophan metabolism from producing kynurenine in *S. aureus* but not in *E. coli*. The reason for different kynurenine levels caused by quercetin is unclear. Additionally, it is not clear how quercetin activated the kynurenine pathway, but this may be connected to the capacity of quercetin to cause oxidative stress. Future studies are warranted to provide a clearer picture. The oxidative modulation of L-tryptophan to produce kynurenine (a degradation product of L-tryptophan) is, however, associated with oxidative stress [27,28,29]. Therefore, it is conceivable that quercetin caused oxidative stress which led to the kynurenine pathway activation. The kynurenine pathway activation implies that quercetin could reduce the availability of L-tryptophan and thus restrict bacterial growth.

## 5. Conclusions

Quercetin, a polyphenol and the most abundant flavonol, suppressed the growth of *E. coli* more than it did that of *S. aureus*. Owing to the high antioxidant property of quercetin, the increase in TAC and total thiol level was not unexpected. However, our data support the capacity of quercetin to cause kynurenine pathway activation in *S. aureus*, thereby limiting the local availability of L-tryptophan. Reduced local L-tryptophan concentration might likely deprive bacteria of the required growth nutrients, thus affecting survival. In *S. aureus* and *E. coli*, quercetin also caused lipid peroxidation but not DNA damage. Considered together, the kynurenine pathway activation and possibly the promotion of oxidative stress might be the means through which quercetin restricts bacterial or microbial growth.

## Figures and Tables

**Table 1:** Inhibition zone following treatment of isolates with quercetin.

Microbial Isolates	Quercetin (mm ± Standard Deviation)	Amoxicillin (mm ± Standard Deviation)
***S. aureus***	12 ± 0.9	19.5 ± 0.5
***E. coli***	14 ± 0.5	25.5 ± 3.5
***P. aeruginosa***	21.5 ± 1.5	33.5 ± 1.5
***K. pneumonia***	25 ± 1.0	-
***B. subtilis***	20 ± 1.0	16 ± 1.0
